# Optimisation of a machine learning algorithm in human locomotion using principal component and discriminant function analyses

**DOI:** 10.1371/journal.pone.0183990

**Published:** 2017-09-08

**Authors:** Maria Bisele, Martin Bencsik, Martin G. C. Lewis, Cleveland T. Barnett

**Affiliations:** School of Science and Technology, Nottingham Trent University, Nottingham, United Kingdom; University of Illinois at Urbana-Champaign, UNITED STATES

## Abstract

Assessment methods in human locomotion often involve the description of normalised graphical profiles and/or the extraction of discrete variables. Whilst useful, these approaches may not represent the full complexity of gait data. Multivariate statistical methods, such as Principal Component Analysis (PCA) and Discriminant Function Analysis (DFA), have been adopted since they have the potential to overcome these data handling issues. The aim of the current study was to develop and optimise a specific machine learning algorithm for processing human locomotion data. Twenty participants ran at a self-selected speed across a 15m runway in barefoot and shod conditions. Ground reaction forces (BW) and kinematics were measured at 1000 Hz and 100 Hz, respectively from which joint angles (°), joint moments (N.m.kg^-1^) and joint powers (W.kg^-1^) for the hip, knee and ankle joints were calculated in all three anatomical planes. Using PCA and DFA, power spectra of the kinematic and kinetic variables were used as a training database for the development of a machine learning algorithm. All possible combinations of 10 out of 20 participants were explored to find the iteration of individuals that would optimise the machine learning algorithm. The results showed that the algorithm was able to successfully predict whether a participant ran shod or barefoot in 93.5% of cases. To the authors’ knowledge, this is the first study to optimise the development of a machine learning algorithm.

## Introduction

Gait analysis attempts to describe the characteristics of human locomotion [1, 2]. From a clinical perspective, it is often used to assess the effects of conditions on gait, such as cerebral palsy [3], and lower limb amputation [4], and has led to improved diagnoses, enhanced treatment recommendations and enabled the evaluation of treatment outcomes [2, 5]. During gait analysis, data acquisition tools such as motion capture systems, force plates and electromyography are used to assess the biomechanical and physiological characteristics of gait. Kinematic and kinetic data, such as joint angles and ground reaction forces (GRF) are often reported, however subsequent analysis, such as inverse dynamic calculations, can be conducted to assess further aspects of gait such as joint moments and powers [6, 7]. These variables are often presented in temporal waveforms or time-series throughout the gait cycle [7, 8].

As data acquisition tools used to collect gait data and the subsequent procedures for calculating novel variables continuously advance, they provide an ever increasing volume of data [7, 8]. This presents a hindrance to clinicians and researchers when trying to interpret this data and/or forming clinically useful information [9]. A widely used approach to analyse and interpret movement data is through the description of graphical profiles of temporal waveforms and reinforcement of this information through the use of discrete variable extraction [8, 9]. Research has also attempted to summarise waveforms of gait data using indices and summary scores [10–13]. However, the interpretation of graphical profiles may be researcher-dependent, meaning results may differ between patients and/or laboratories. Similarly, the choice of data collected is dependent upon equipment availability, and researchers’ selection of variables. This can be an issue in a clinical environment as variables assessed in a patient may not be the cause of a problem, thus results may show no significant difference with the primary problem remaining undetected and untreated. Therefore, data collection, analysis and interpretation are somewhat subjective and prone to researcher bias.

Gait data is also governed by a set of well-defined characteristics namely 1. high dimensionality, 2. time-dependence, 3. high variability, and 4. nonlinear correlations residing between the variables independently measured [14, 15]. During data processing, these characteristics are important to consider, however, while previously mentioned methods such as gait indices, summary scores and the extraction of discrete variables may be adequate to provide enough information in some investigations, in others they may not be suitable since temporal information is lost [16]. For example, measurements are often repeated for a given individual in order to account for intra-subject variations. Such variations may result in specific discrete variables that take place at different timings of the measurements for each repeat. These would be neglected in methods where the time-series of the data is ignored/removed. In instances where the time-series of the data is accounted for, careful phasing of the waveforms is required to access the information relating to the absolute gait cycle. Also, certain features reside in the shape of the gait time-series, e.g. in pathological gait [8, 17] where distortions result from pathology and thus are not picked up in methods using purely scalar quantities. Recent studies introduced the use of Spatial Parameter Mapping (SPM) technique considering entire gait waveform thus preserving temporal characteristics of data during statistical analysis [18, 19]. In addition, usually, there will not be fixed linear relationships between variables and each variable may affect or be affected by one or more other variables differently [14, 17]. Therefore, the problem of high dimensionality will often prompt the researcher to identify the variable(s), also known as informative features that change the most as the most important/relevant. However, changes in this variable may reflect the summation of multiple smaller changes in other variables, some of which are sometimes unmeasured, which scale it and infer it is the underlying cause for the difference in gait. For example, when reporting net muscle moments, the individual muscle forces, which drive these moments, are not routinely reported. Hence, there is a need for effective quantification methods to reduce dimensionality sets and establish relationships between variables while retaining temporal information.

Principal Component Analysis (PCA) is a well-established multivariate analysis used for data reduction and sometimes classification, first applied to human locomotion by Deluzio et al. [8]. Typically, it has been used in lieu of statistical comparisons to classify data while maintaining the variance in the structure of the original data. It has had many applications in the comparison of able-bodied gait and pathological gaits such as amputee gait [20–23], osteoarthritis gait [16, 24] and Parkinsonian gait [25–27]. Linear PCA is a strong algorithm that is able to detect patterns in certain structures, without user supervision. Therefore it deduces informative features for classification from the structures without direction, lead purely by the variance within the data [28] and it seeks the most efficient representation of the original database using Principal Components (PCs). Discriminating factors of interest to the user may go undetected if they are of small magnitude, and/or if they end up being shared between too many Principal Components. Thus, to identify subtle discrimination features that exist between experimental groups, a supervised algorithm such as Discriminant Functional Analysis (DFA) is required. The multivariate statistical method of DFA seeks out linear combinations of the input variables, the PCA scores in our case, in order to best discriminate between groups and therefore highlight differences in the detailed structure of the data where PCA only discriminates between gross structures [28]. Together the combination of unsupervised and supervised numerical search algorithms create a powerful machine learning algorithm, which refers to the ability of a device to independently conduct discrimination on a database without the input of a researcher [22, 29]. In a clinical setting, a machine learning algorithm can provide an objective method to conduct analyses and thus eliminating researcher bias when assessing gait data [29]. Previous studies have used machine learning algorithms to identify gait differences between young and older adults [30–33], males and females [34], pathological and non-pathological gait [22, 24, 29, 35, 36] and high discrimination results have been obtained such as 91.7% or 95.8% between older and younger individuals [30, 31, 36], between males and females with 98–100%, and 100% between pathological and non-pathological gait [22]. However, in less challenging environments, where differences between distinct groups (e.g. males vs. females) are more easily detected, previous studies have not considered how the quality of data used to train the machine learning algorithm, can deteriorate the quality of the outcome. Different individuals will exploit features in different manners and will not necessarily train the computer in the way that will work best for other individuals that the automated system will analyse later in its predictive stage. At the training stage, it is therefore important to supply the algorithm with a training database that has been carefully selected for its aptitude to reveal the best, highly generic, discriminating features. Furthermore, the PCA algorithm outputs information in a new space defined by a set of axes necessarily orthogonal to each other, causing features of interest too often reside within more than one PCA score. How successful the DFA algorithm will be in pulling out the important discriminating features will, therefore, depend on the extent to which these features have blurred out onto multiple PCA axes.

Therefore, the specific individuals that have been chosen and used to develop the training database for the machine learning algorithm will strongly affect its predictive abilities. Using increasingly larger database for the training stage is of no help, as the feature blurring into multiple PCA scores will only become worse, which we will attempt to address in the current study.

It is, however, possible to work with a smaller sample and try to optimise predictive accuracy by implementing an iterative process where the individuals contributing to the training stage are systematically permuted. In our current study, we find hidden groupings using all possible permutations for resampling our database. We develop, demonstrate and evaluate such a resampling procedure for processing human locomotion data, using power spectra of temporal waveforms as an efficient compromise to keep temporal information without the need to either phase the data or compute user-defined discrete variables.

## Methods

### Participants

A convenience sample of twenty recreationally active participants (14 males and 6 females; age 24±4 years; height 1.75±0.086m; mass 72.0±8.5 kg) were drawn from the University community. These individuals who had no lower limb pathologies, and were free of injury during the time of the study, provided informed consent to participate. Ethical approval was granted by the Nottingham Trent University Ethics Committee (Humans).

### Experimental design

The study investigated participants under two different experimental conditions; running with (shod) and without shoes (barefoot) with trial conditions being counterbalanced between participants.

### Data acquisition

Participants completed all activities wearing their own shorts and running shoes. To measure kinematic data, 36 spherical 14mm, retro-reflective markers were placed directly onto the skin or clothing using bi-adhesive tape. Markers were attached bilaterally and used to define trunk [37] and lower limb segments [38].

Participants warmed-up with a five minute run on a treadmill at self-selected speed. After the warm-up, participants proceeded to the experimental trials, which required them to run at self-selected speed along a 15m runway making contact with a force plate. This process was repeated until five successful trials (force plate contacts) had been recorded for each condition. Once completed, the process was repeated for the second condition of experimental trials. Ground reaction force (GRF) was measured at 1000Hz using one floor-mounted strain gauge force plate (AMTI, Watertown, MA, USA) and kinematics were measured at 100 Hz using a nine-camera motion capture system (Qualisys, Gothenburg, SE).

### Data pre-processing

The raw marker trajectories and force data were exported as .c3d files and processed in Visual 3D v5 (C Motion, Inc., Germantown, MD, USA). Kinematic data were interpolated using a cubic-spline algorithm with kinematic and GRF data being subsequently filtered using 4^th^ order, zero-lag Butterworth low-pass filters with 6Hz and 30Hz cut-off frequencies respectively. Medial and lateral landmarks defined anatomical frames from which segment co-ordinate systems were defined following the right-hand rule [38]. A flexion-extension, abduction-adduction and longitudinal cardan rotation sequence was used to define the order of rotations to calculate joint kinematics. Gait events of heel strike (HS) and toe off (TO) were determined using GRF data and data all were normalized to 100% gait cycle.

Joint angles (°), joint moments (N.m.kg^-1^) and joint powers (W.kg^-1^) for the hip, knee and ankle joints, as well as the GRF (multiples of body weight; BW) were computed using Visual 3D (C-Motion, Inc, Germantown, USA). Results were reported in all three anatomical planes, thus thirty temporal waveforms were reported for a single trial, which was considered to start when the right limb hits the force plate at heel strike and finished at the consecutive heel strike on the same limb. Processed data were exported from Visual3D in .c3d files and individual signals from the .c3d files were imported to MATLAB^®^ R2013a (MathWorks Inc., MA, USA).

### Machine learning algorithm

In our study, we optimise a specific machine learning algorithm that would distinguish between two experimental conditions of barefoot and shod running. The development of the machine learning algorithm, using PCA and DFA was done in three stages of dimensionality reduction, informative feature extraction and classification. Prior to conducting PCA or DFA, the data were linearly interpolated to the same digital length to allow the power spectrum (modulus of FFT) to be computed for all variables. This allowed us to remove the absolute phasing of kinetic and kinematic waveforms which would affect the discrimination process since any error in the phase correction required to obtain the absolute gait cycle would be spuriously identified as a discriminating feature between the trials, compromising the outcome of the machine learning algorithm. Apart from the absolute phasing of different frequency components of the data, the rest of the temporal information of the waveforms is kept intact in the power spectra.

An input matrix *M* was established which contained the power spectra of the kinetic and kinematic waveforms extracted from each experimental trial. The matrix was ordered as follows: for each subject five trials of each condition existed (twenty subjects resulted in 200 trials) and every trial was made of 30 columns with 50 row vectors, where each column represented a variable and each row vector represented the frequency of the 3D coordinate measure of the variable. The input matrix *M*, originally 3D with 200 x 30 x 50 points, was rearranged to be 2D, with 200 x 1500 points, in order to undertake the PCA on a collection of 200 trials each comprising of 1500 points.

First, the data were summarised using PCA and thus high dimensionality was reduced from the original 1500 points (for each trial) to 8, 10 or 12 points. Our numerical analysis was made immune to overfitting artefacts originating from the over-exploitation of small details, by choosing the highest explored rank (12^th^) well below the one still carrying information (20^th^) (see supplementary material S3 Fig). Principal Component Analysis (PCA) is an orthogonal transformation turning dependent variables of a multivariate database to a small set of independent new variables or Principal Components Z, which are used to represent the variance observed in the original variables X [14]. Components Z make up the columns of the correlation/covariance matrix (covariance in the current study) and are eigenvectors, also referred to as loading vectors. The Principal Components (PCs) are ordered in terms of decreasing variance such that the majority of variation in the data can usually be described by the first couple of PCs and therefore the remaining PCs can be ignored reducing the dimensionality of the data which commonly reduces the noise in the input data *X*. However, depending on the research question this may not hold true and medium or lower ordered PCs may provide the necessary information rather than higher ordered PCs [39].

Using the reduced database, DFA was further applied to a selection of PC scores (up to the eighth, up the tenth and up to the twelfth score), in order to identify generic discriminating features between the two experimental conditions, and cluster the data as required by the goal of the study (shod versus barefoot). Discriminant Function Analysis (DFA), also known as Linear Discriminant Analysis (LDA), is a statistical analysis which works to attain the maximum discrimination between classes. To achieve maximum separation, the ratio of inter-class and intra-class variance for any given database is computed. This results in linear class boundaries thus grouping the various class clusters in a given subspace [40].

**Fig 1:**
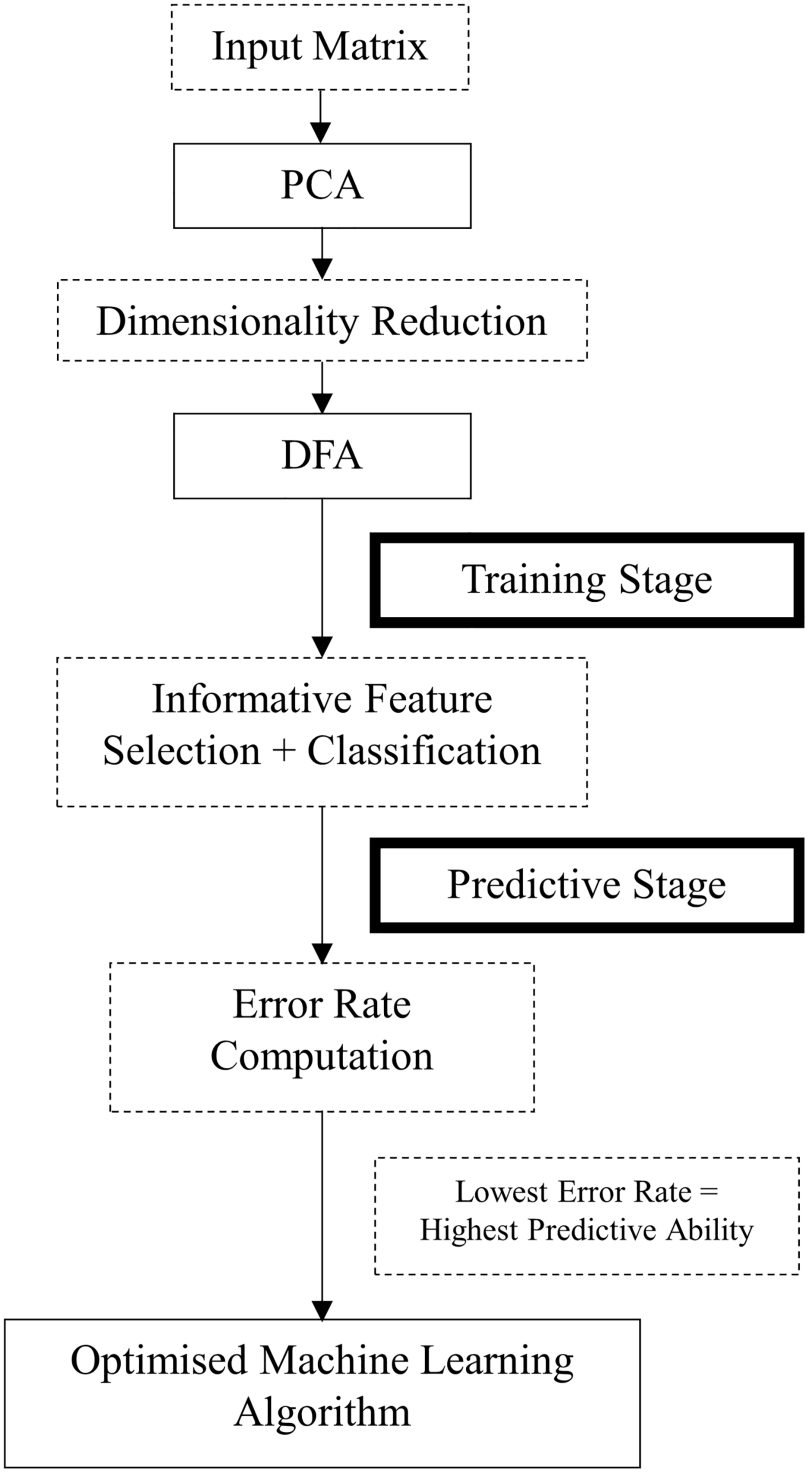
Flow-chart of the development of the machine learning algorithm.

All previous stages were combined to develop the machine learning algorithm which is also referred to as a predictive algorithm, when applied to data that did not contribute to the learning stage. To optimise its development, the process was divided into two stages of training and predictive (see Fig 1). During the training stage, data from ten participants was used to direct the search for generic features and identify which of these provided the greatest discrimination between the two experimental conditions. During the predictive stage, data of the remaining ten participants that had not contributed to the training of the machine learning algorithm were used to assess whether it could correctly assign data to the group with the same generic features.

### Optimisation process

**Fig 2:**
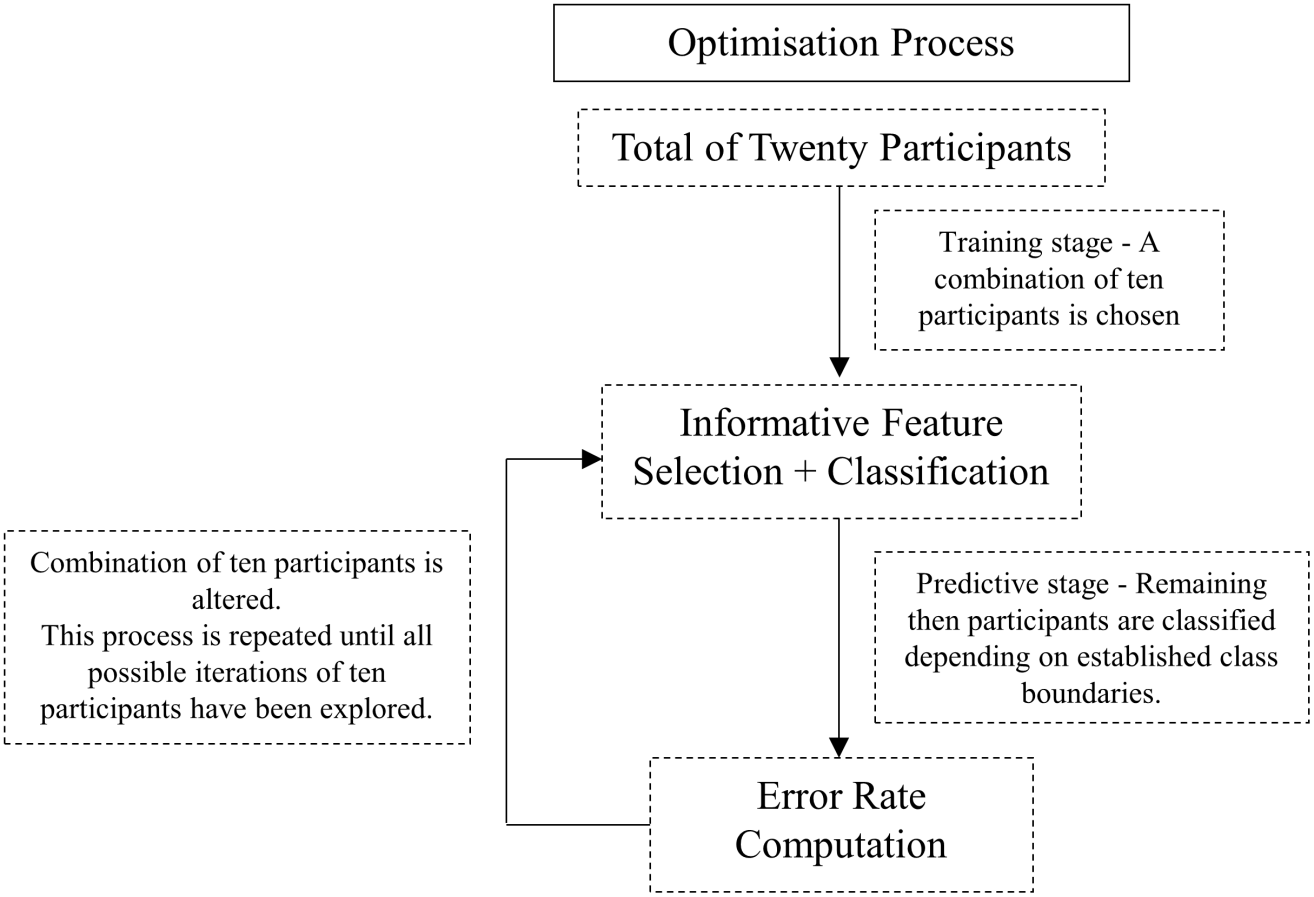
Flow-chart of the iteration process used to optimise the machine learning algorithm.

**Fig 3:**
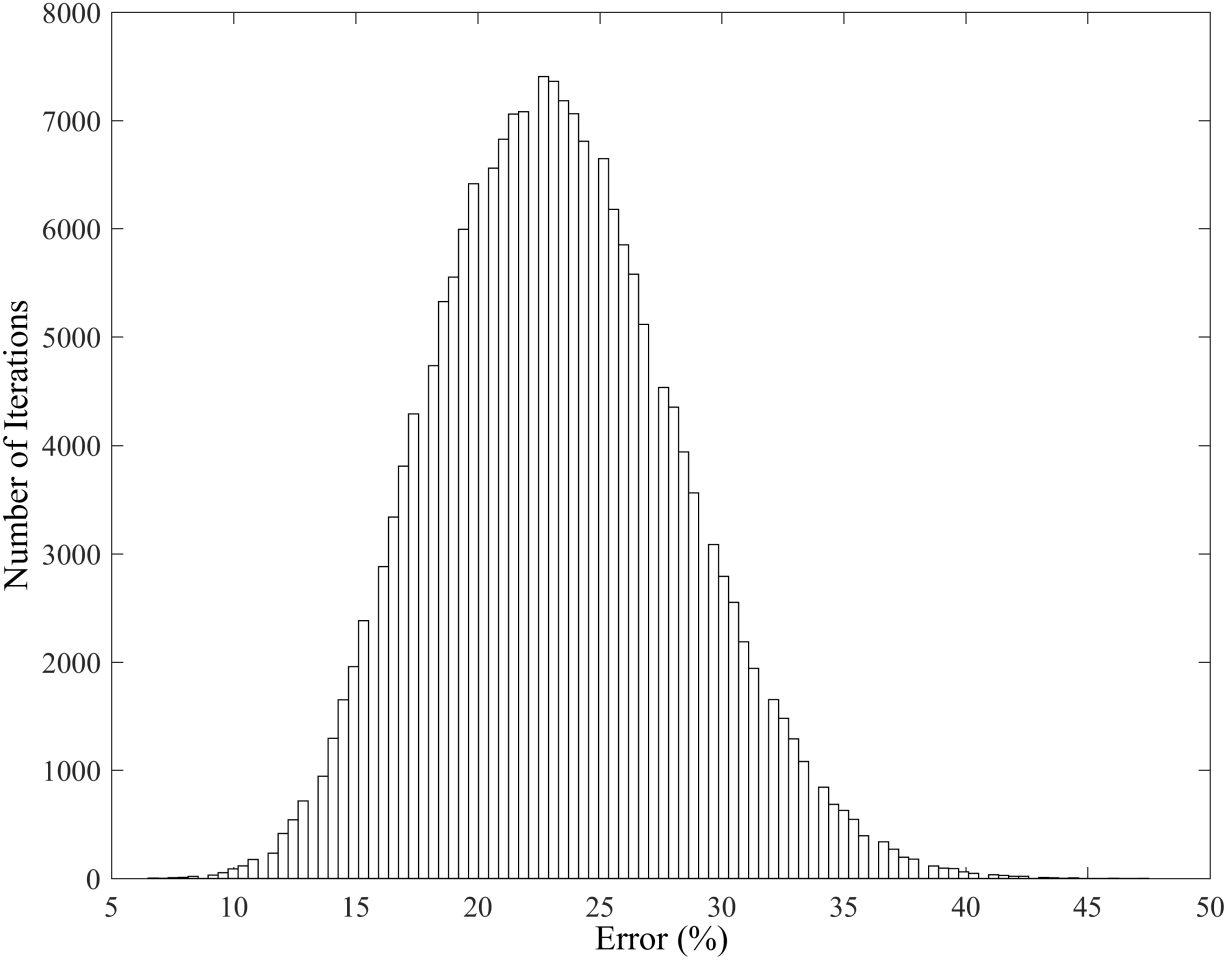
Histogram indicating the error rates of discrimination for each individual iteration. An iteration consisted of a different combination of 10 participants out of 20 for each the training and predicted database. The error is the percentage of variables that end up in the wrong category (shod or barefoot).

To identify which ten participants would best train the algorithm and result in the best overall classification, allowing the most predominate, generic discriminating features to be identified between the two experimental conditions, all possible combinations of 10 out of 20 participants were explored (see Fig 2); a total of 184,756 iterations were identified and assessed therefore optimising the algorithm. An error rate was computed for each individual iteration (see Fig 3) and the one yielding the combination of participants with the lowest error rate revealed the strongest generic discriminating features.

The error rate was calculated as follows: each trial was projected onto a two-dimensional DF space, yielding a set of two DF scores. In this space, the coordinates of the two centroids were calculated, and for each trial, the Euclidean distances to both centroids were further calculated. The ratio of these two distances was used to assess whether the trial ended up in the ‘shod’ or ‘barefoot’ category, with a value of 1 corresponding to the threshold dictating the membership. The trials ending up with the incorrect membership were expressed as a percentage error rate, over all the 200 trials (20 individuals each undertaking 5 shod and 5 barefoot runs).

### Classification evaluation and performance measure

A confusion matrix has been used to evaluate the performance of the machine learning algorithm. In a two classes problem, positive and negative, as it is the case in the current study, there are four possible outcomes of classification, namely true positive (TP), false negative (FN), true negative (TN), and false positive (FP). In the current study, positive instances relate to shod running trials and negative instances relate to barefoot running trials. The sensitivity and specificity (see Eqs 1 and 2) respectively refer to positive and negative instances which have been correctly identified.

Sensitivity (SEN) or true positive rate (TPR):

$$\text{sensitivity}=\frac{\text{true positives}}{\text{true positives}+\text{false negatives}}$$

$$TPR=\frac{TP}{P}=\frac{TP}{TP+FN}$$ (1)

Specificity (SPC) or true negative rate (TNR):

$$\text{specificity}=\frac{\text{true negatives}}{\text{true negatives}+\text{false positives}}$$

$$TNR=\frac{TN}{N}=\frac{TN}{TN+FP}$$ (2)

## Results

The outcome of all possible iterations, as shown in the histogram of Fig 3, indicates that the error rates of trials which could not be correctly classified ranged from 6.5% to 47.5% and the majority of iteration were identified to have an error rate of 22.5%. This clearly demonstrates how much the algorithm can be helped by careful selection of the training database. As previously mentioned the lowest error rate indicated the highest predictive ability, and thus the iteration corresponding to 6.5% was used as the input for the optimised machine learning algorithm.

**Fig 4:**
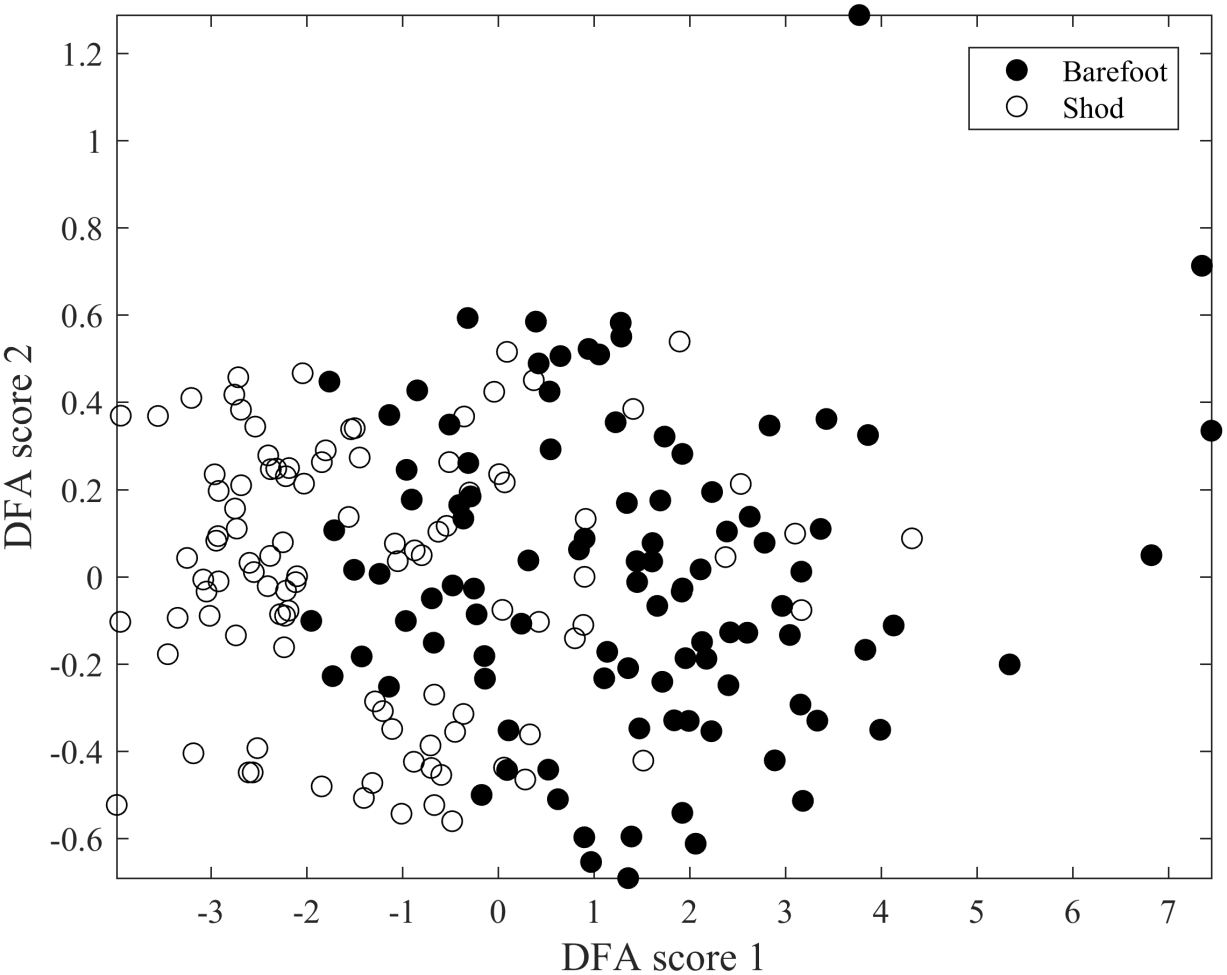
Unoptimised result, showing data following discrimination undertaken on the entire collection of measurements, both for PCA and DFA, resulting in an error of 24%.

Increasing the rank of the PCA scores fed to the DFA algorithm from 8 to 12 did not improve the outcome, and the data we show was obtained using 10 PCA scores. Using the entire database as the training database for the discrimination exercise yielded an error rate of 24% as seen in Fig 4.

**Fig 5:**
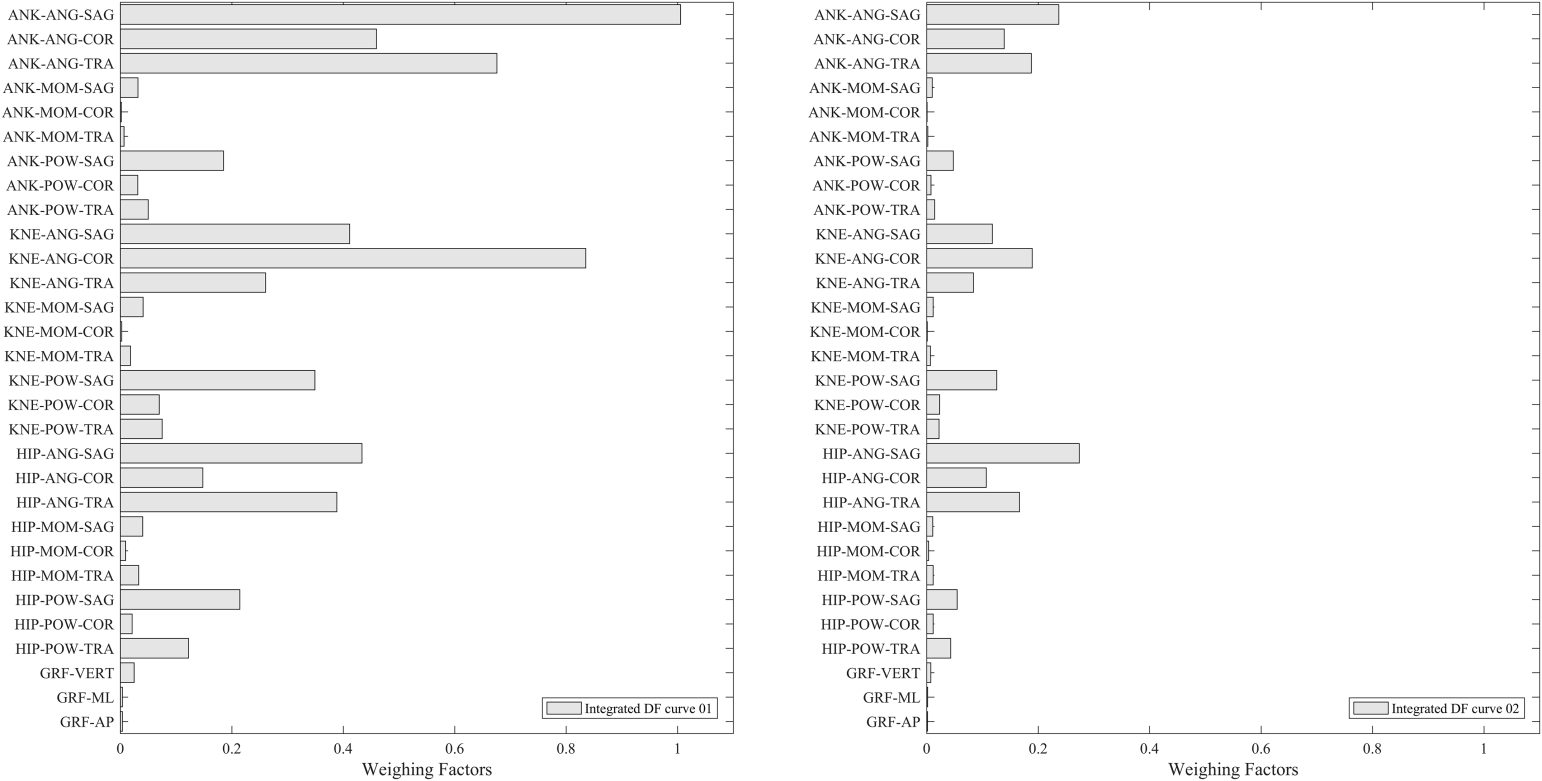
DFA discrimination figure showing two bar charts where each bar is equivalent to a measured variable from a DF curve, integrated over all spectral frequencies. Abbreviations are knee (KNE), ankle (ANK), angle (ANG), moment (MOM), power (POW), anterior-posterior (AP), medial-lateral (ML) and vertical (VERT).

**Fig 6:**
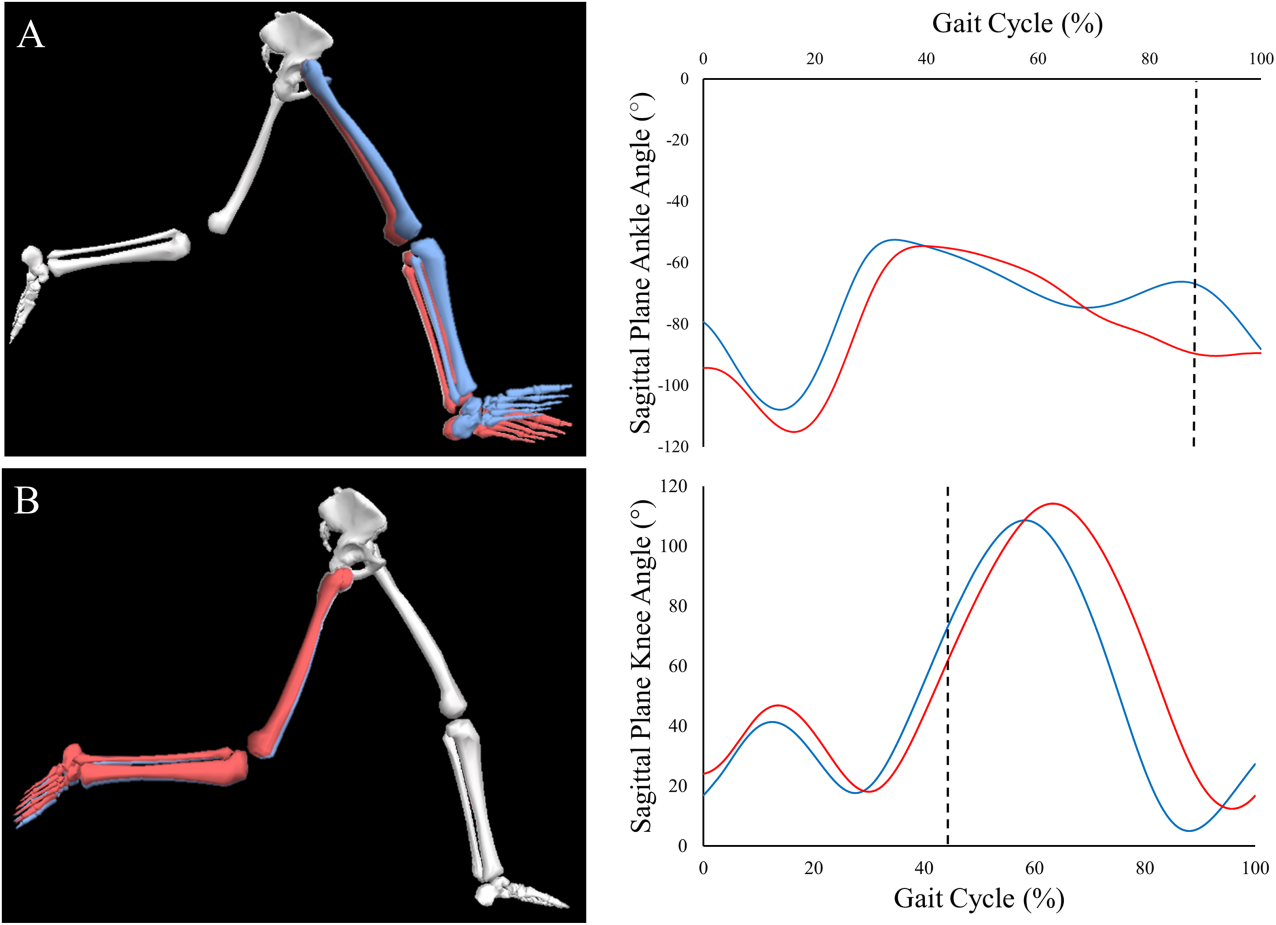
An illustrative representation of exemplar highly discriminating (A—sagittal plane ankle angle) and lower discriminating (B—sagittal plane knee angle) variables from a single participant during both shod (red limbs and lines) and barefoot (blue limbs and lines) running. Dashed lines represent the instance in the gait cycle that the illustrations are taken from.

The optimum iteration was further used to identify the most discriminating features between the two experimental groups of barefoot and shod running using DFA as illustrated in Fig 5. The different bar charts correspond to different DF curves integrated over all spectral frequencies (full frequency-resolved DF curves are shown in the supplementary material S1 Fig), where each bar represents a variable. The fact that they are dissimilar justifies the benefit of undertaking the discrimination in two dimensions rather than one. The length of each bar emphasises the weight factors of individual kinetic and kinematic variables (averaged over all frequencies). Long and short bars had a high and a low contribution to the discrimination process, respectively. Since the analysis was conducted for thirty variables, there are thirty bars for each integrated DF curve. Variables corresponding to individual bars have been ordered, in decreasing order of contribution, and displayed in supplementary material S2 Fig. High contribution variables included ankle angle and power in the transverse plane, ankle angle in the sagittal plane and ankle moment in the coronal plane whereas low contribution variables corresponded to knee angle and moment in coronal plane, and medial-lateral and the anterior/posterior GRFs. An example of a highly discriminating, and a low discriminating variable is shown in Fig 6.

**Fig 7:**
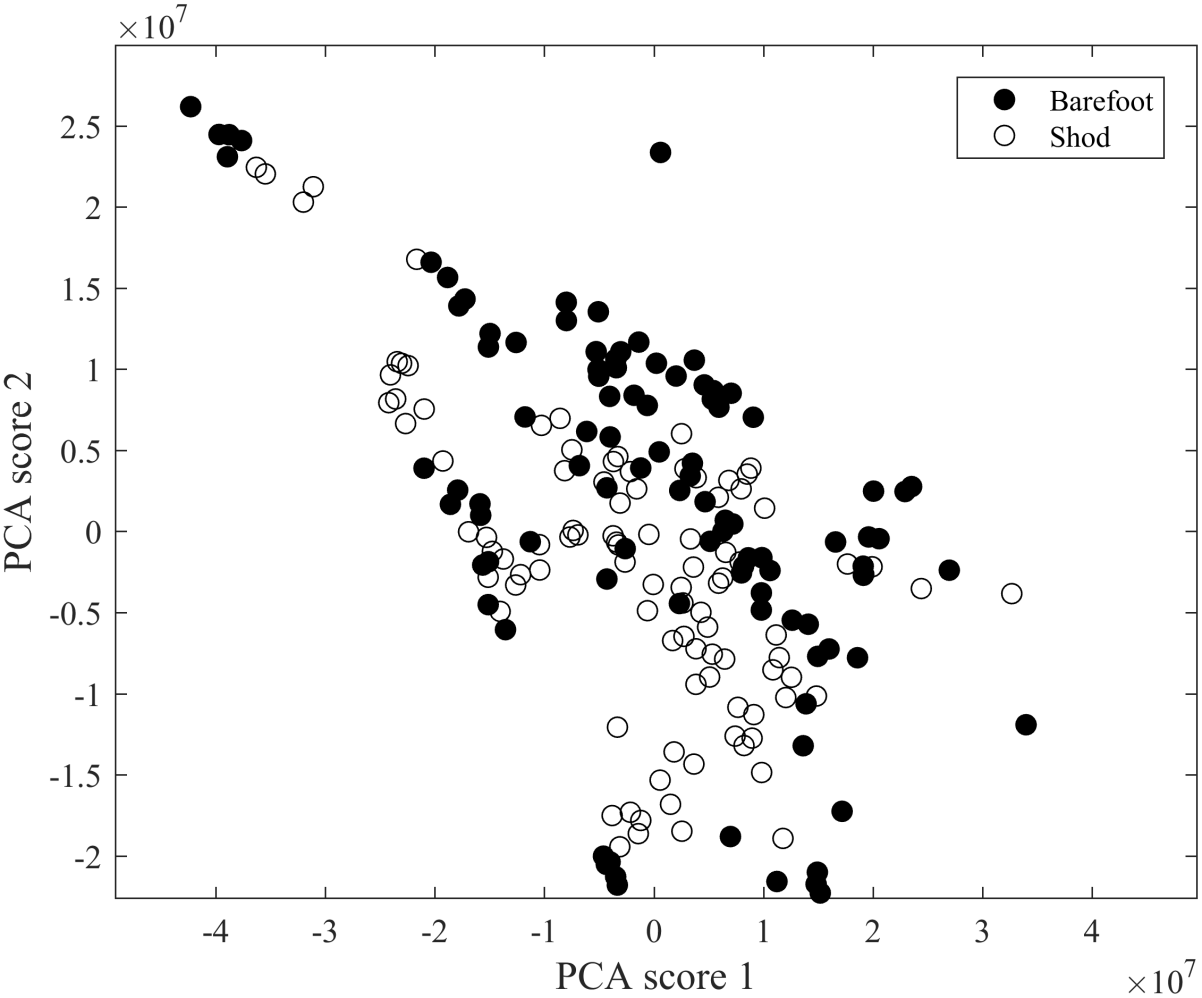
Outcome of PCA following classification. Each dot represents a trial of a participant and since each participant has conducted 10 trials (5 shod and 5 barefoot) and there was a total of 10 participants the figure illustrates the discrimination of 100 trials.

The outcome of the PCA search (Fig 7) alone results in severely overlapping clouds, demonstrating the fact that the discrimination that was sought for is not residing in the main deviations found in the data, illustrating the challenging nature of the conditions of interest. Instead, the discrimination required (shod/barefoot) resides in subtle details of the spectra, necessitating the second stage numerical search, DFA, to be applied to the data after reduction of PCA. We also undertook visual examination of both the time courses and the spectra of our ‘barefoot’ and ‘shod’ conditions, and no clear common discriminating patterns emerged in spite of careful inspection.

**Fig 8:**
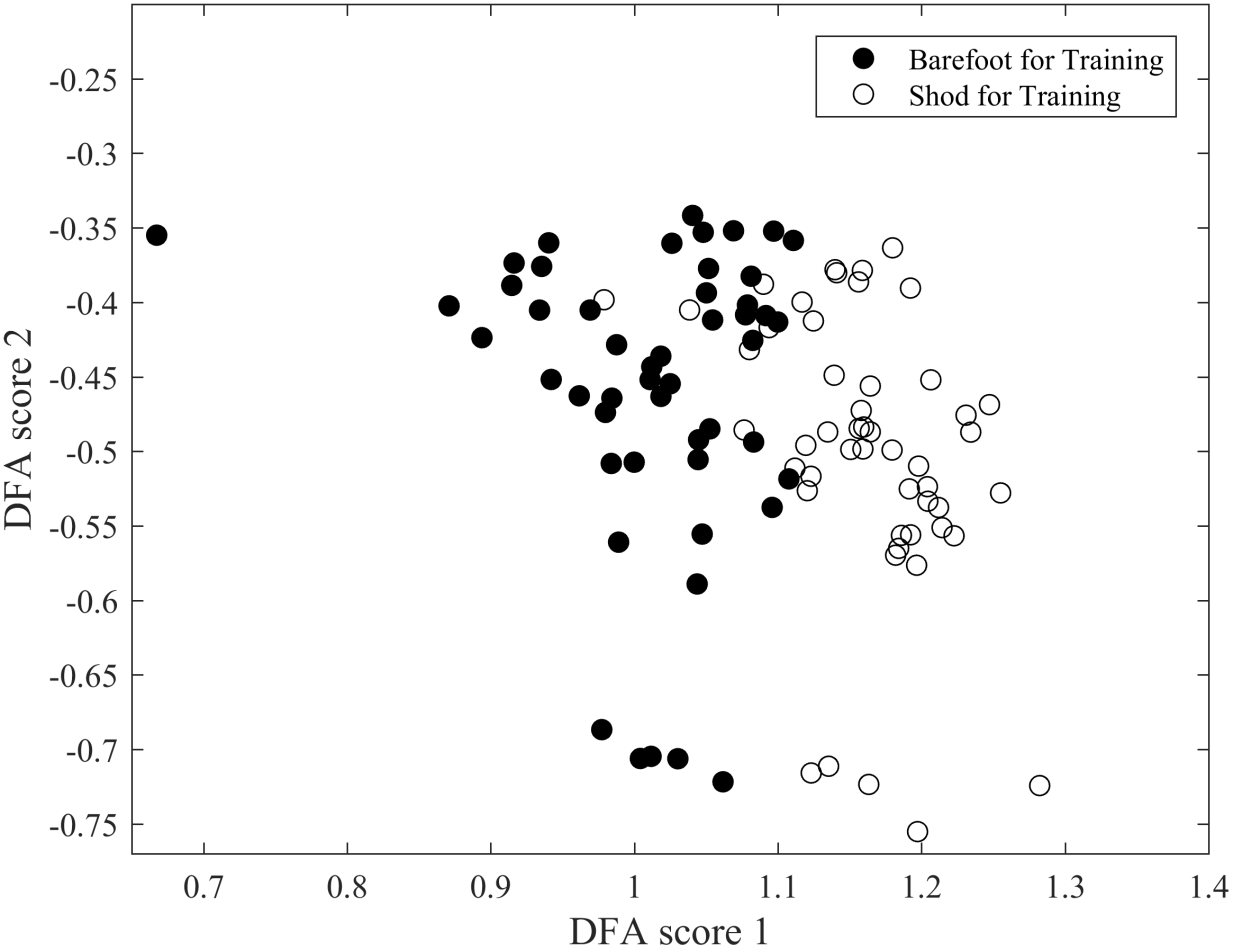
Outcome of training database following discrimination, from the 10 participants with the smallest error in prediction.

**Fig 9:**
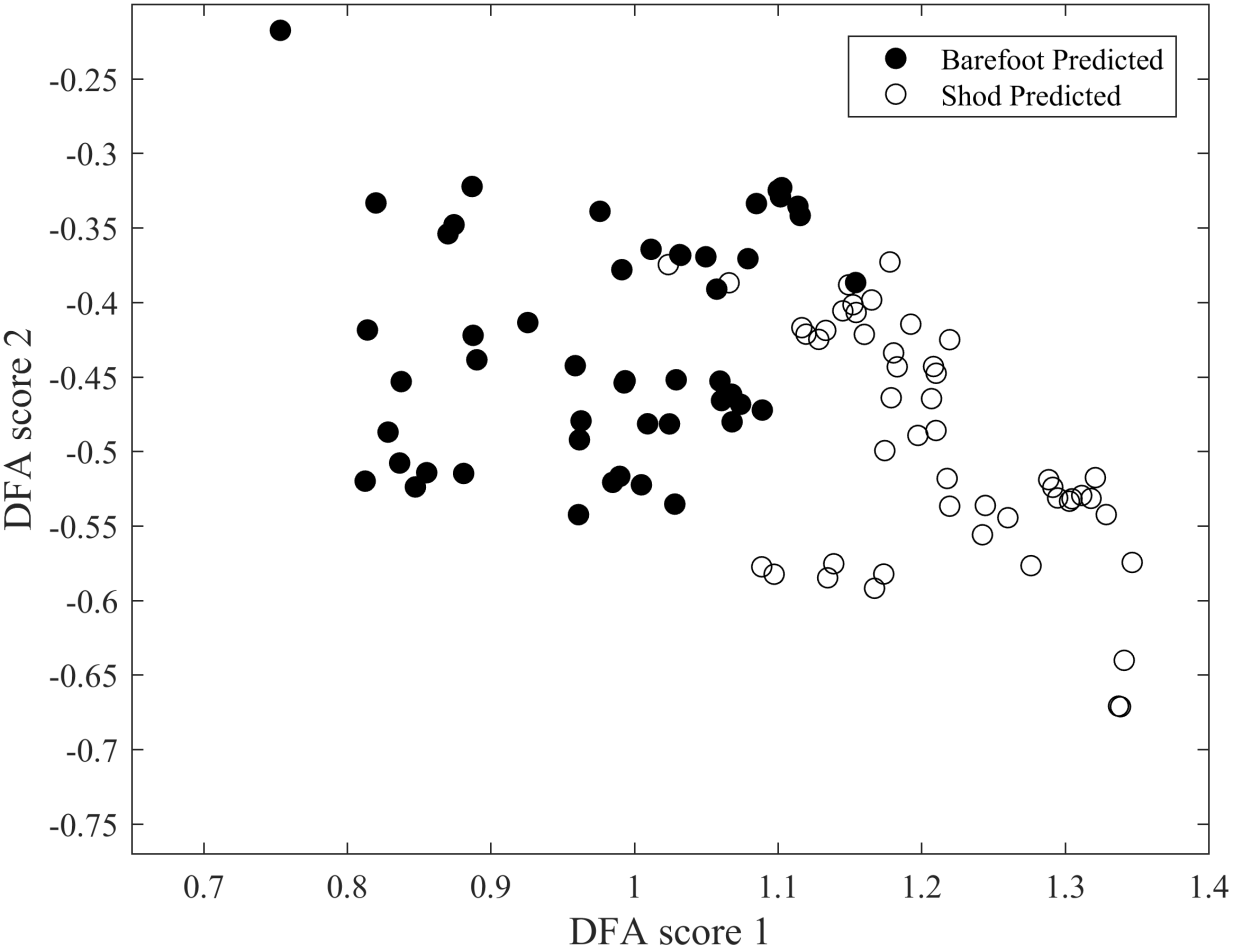
Outcome of discrimination for the 10 participants not used to generate the machine learning algorithm. The scatter slightly greater than in Fig 3, but an excellent reliability in terms of correct discrimination.

**Fig 10:**
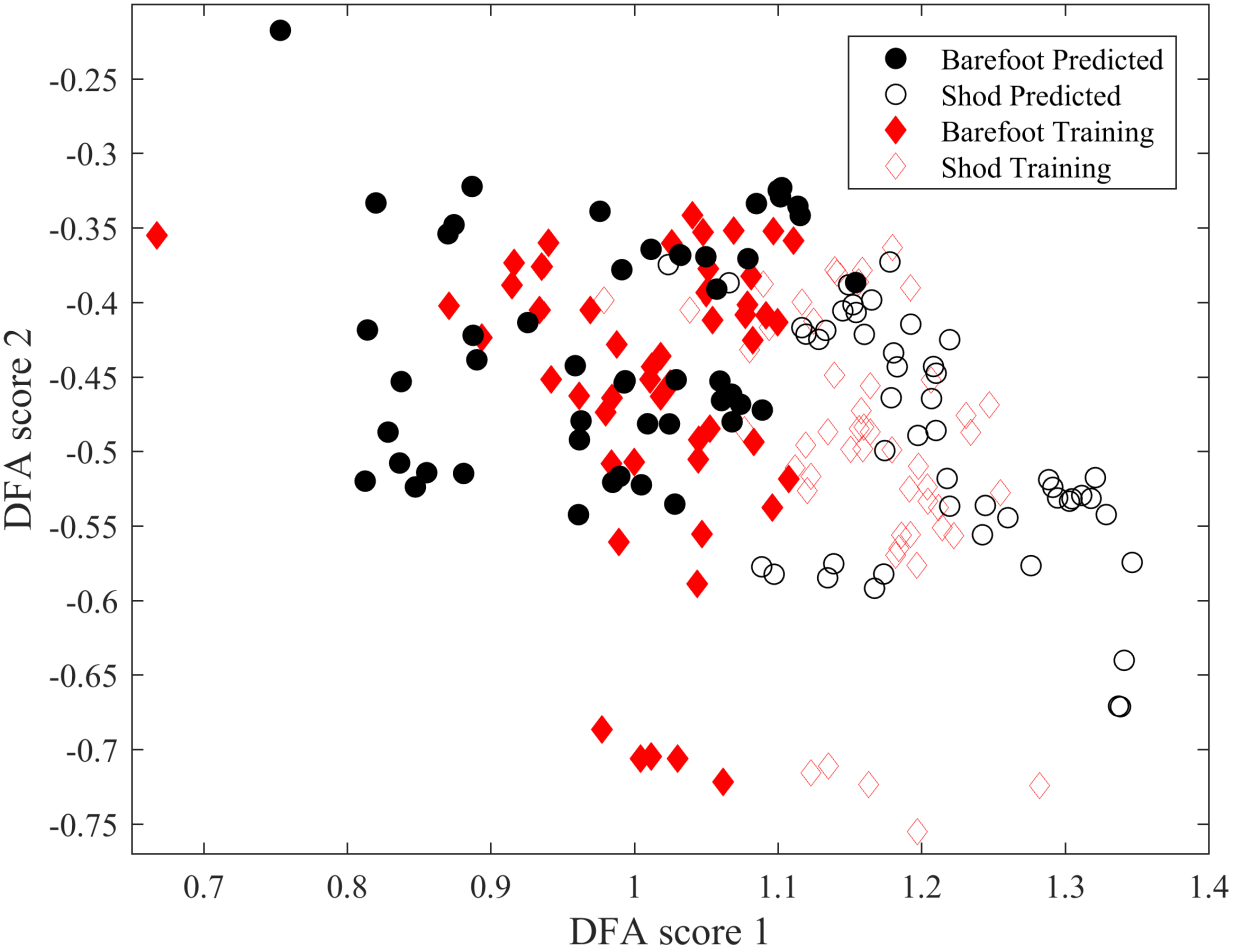
Combined display of trained and predicted data following discrimination.

The quality of the discrimination obtained with our optimised DFA is illustrated in Figs 8, 9 and 10. The quality in discrimination is evidenced by the minimal amount of overlap between the two conditions; two well-discriminated groups will not occupy the same space. The outcome of the training database alone, used to develop the algorithm is shown in Fig 8. Once developed the predictive ability of the algorithm was assessed as illustrated in Fig 9. It can be seen that even though there is a slightly greater scatter in the predictive outcome it does not compromise the quality of discrimination: when the software has been given a chance to be trained with the ideal training data base, Figs 8 and 9 suggest that the computer is further able to correctly discriminate those individuals that have a rather ‘unique’ or ‘rare’ way to run shod and barefoot. Combining both the outcomes from the training database and the predictive data (Fig 10), it is clear that both experimental conditions of barefoot and shod running are clustered in separate clouds which are shifted to the left and right side respectively, with minimal overlap between the two clouds and a slight vertical slant between the two centroids. The overlap is representative of 6.5% of the trials which could not be correctly discriminated (5% and 8% overlap respectively when considering the predicted data only, and the training data only). The discrimination occurs mostly horizontally with a slight angle indicating that the discrimination is mostly achieved through the DF score 1. Projection onto a higher dimensional space did not yield any significant discrimination. The classification evaluation reinforces these results and shows that sensitivity i.e. true positives (shod and truly identified as shod) would be correctly identified in 90% of cases and specificity i.e. true negatives (barefoot and truly identified as barefoot) would be correctly identified in 91%.

## Discussion

In our study, we optimised the predictive accuracy of a specific machine learning algorithm to distinguish between two experimental conditions of barefoot and shod running. This was done by implementing an iterative process, where the individuals contributing to the training stage were systematically permuted, using an iterative process to explore all possible iterations of 10 participants out of 20 in order to identify generic discriminating features between the two experimental conditions. The optimised algorithm yielded a high discrimination accuracy of 93.5%, typically 17.5% higher than when using a standard analysis.

We achieved high improvement in the software’s performance by using half of our data for training, and the other half for prediction. In instances where the machine learning algorithm is facing the challenge of a mixture of highly ‘generic’ and highly ‘singular’ trials in its training database, we suggest that by homing onto the highly generic individuals, at the stage of training the computer, substantial improvements may be achieved over the entire group, including the highly ‘singular’ individuals. The relatively small group size of our study prevents us from estimating the extent to which accidental spurious information may also have been harvested in the process but limiting ourselves to only 10 PCA scores severely limits the likelihood of such phenomena.

An interesting question is whether it might be possible, in any study similar to ours, to identify the best group size to be used when optimising the training. Unfortunately, the extent to which specific volunteers provide a generic enough feature and the extent to which features of interest become spread between several PCA scores will depend on the specific study undertaken so that no general method can be recommended.

For very large studies, one way forward is perhaps to start by following our optimisation procedure with the same group sizes for training and predicting, and then further refine the collection of ‘ideal’ individuals by swapping one of the ten individuals with a new one to see whether improved discrimination could be obtained. This way the collection of ‘ideal’ generic individuals could gradually be further improved. Using a larger sample then presented in the current study would provide the option to validate the machine learning algorithm since individuals who did not contribute to the training and prediction stages could be used. In such large studies, it is also possible to somewhat reduce the effect of a second possible source of overfitting artefact, that coming from (possibly high magnitude) information accidentally helping the clustering and therefore biasing it. It is possible to quantitate and minimise such overfitting artefacts [41] by splitting the individuals who did not contribute to the training into two groups respectively called ‘validation’ and ‘test’ sets. The trained algorithm can be optimised on the ‘validation’ set only, and those iterations yielding a performance much lower on the ‘test’ set can be deemed as suffering from overfitting and dismissed. Unfortunately, such method is not reliable on the relatively small group size of our study, and the high performance of the optimised outcome of our work suggests that we would have reached the same result if we had implemented it, as both ‘validation’ and ‘test’ sets would have benefitted from a similar performance. Our method systematically tests the algorithm’s performance on data that has not contributed (or biased) the learning of the computer and is therefore inherently minimising overfitting artefacts.

Previous studies have achieved high discrimination results however the quality of data used as a training database for the machine learning algorithms have not been considered which in turn affects the reliability of their predictive outcome [22, 24, 29–35]. Factors affecting the reliability of an algorithm include data from a very limited number of participants since classification results can be high, however, they are not necessarily generic [22]. Unlike other published work our discrimination (see Fig 5) is free from artefacts resulting from training the computer with trials carrying rather rare or unique information. Moreover, the context of the experimental protocol influences the results of a discrimination since some experimental groups or conditions are easier to distinguish than others, in particular in instances where the two groups to be discriminated are necessarily formed from different individuals, e.g. young vs. older individuals, normal vs. pathological gait and males vs. females [24, 29–35]. Thus in the development of the current machine learning algorithm, the same heterogeneous sample of participants repeated both experimental conditions. This creates a more challenging environment, when compared to having clearly discrete homogenous groups e.g. healthy vs. pathology, whose data is independent of one another. Therefore, the outcome of the algorithm presented in the current study was more likely to reflect the ability of the algorithm rather than experimental group differences.

Developing a machine learning algorithm using scalar quantities extracted from the waveforms of kinetic and kinematic variables [29, 30, 32, 33] could result in the dismissal of important temporal data, thus power spectra of full waveforms have been employed [24, 35, 36] since each individual feature provides complementary information [42]. In the current study, the training database used to conduct a numerical search using PCA and DFA included the spectra of thirty full temporal waveforms of kinetic and kinematic variables for each trial thus the entire waveform of a variable was taken into consideration.

In previous studies, ankle kinematic and kinetic variables such as plantar flexion [43,44] have been shown to differ between barefoot and shod running gait [43–45]. Studies have also reported limited differences between barefoot and shod runners in GRFs [46, 47]. Although not the specific focus of the current study, the results of the current study confirmed these findings, suggesting that these variables represent the key differences between shod and barefoot running gait. However, unlike previous research, the choice of variables selected in our study as an input to the machine learning algorithm were generic biomechanical features, and not specifically selected, thus reducing researcher bias and reflecting the true ability of the algorithm to identify the generic discriminating features.

The development of the machine learning algorithm described has many important applications in both clinical and research settings. In clinical settings, it allows for a more comprehensive and consistent assessment process across patients by utilising a wider range of data whilst simultaneously eliminating researcher bias. Furthermore, since all discriminating features are identified, in both a clinical and research setting, it will prevent important factors being neglected and ensure accurate and reliable diagnosis. This will enable analysis methods to be more objective, consistent and reliable across institutions.

## Conclusion

A specific machine learning algorithm, using composite PCA and DFA, was developed using power spectra of temporal waveforms to successfully identify barefoot and shod running gait. The predictive accuracy of the algorithm was optimised in a challenging environment by implementing an iterative process. All discriminating features between the two experimental groups were identified and a strong machine learning algorithm was developed with a 93.5% accuracy in discriminating between conditions. This method can be implemented, to find informative features when the sample size is small and heterogeneous, as common in gait analysis.

## Supporting information

S1 Fig Full DFA spectra. The right-hand side vertical axis is valid for variables 1 to 3, whilst the left-hand side one is valid for all remaining variables. Note that frequencies above 150 Hz do not contribute to the discrimination and the high importance of ultra-low frequencies. In some instances (e.g. variables No. 15 and 24), frequencies as high as 90 Hz contribute to the discrimination. In the main manuscript, the data shown in Fig 4. was obtained by integrating the absolute values of the spectra shown here, over all frequencies. The data shown in Fig 10. is obtained by cross-correlating the spectra shown here with the spectra coming from the raw variables (cross correlating any raw variable spectrum respectively with DF spectra 1 and 2 provide the corresponding DF score 1 and 2).
(TIF)

S2 Fig Thirty variables in decreasing order of contribution to the discrimination between barefoot and shod runners. (TIF)

S3 Fig PC score amplitude drop, with PC score rank, showing that up to 20 PC scores carry relevant information. (TIF)

## Abbreviations

DF: Discriminant Function
DFA: Discriminant Function Analysis
FN: False negative
FP: False positive
FFT: Fast Fourier Transform
GRF: Ground reaction forces
HS: Heel strike
LDA: Linear Discriminant Analysis
PCs: Principal Components
PCA: Principal Component Analysis
SEN: Sensitivity
SPM: Spatial Parameter Mapping
SPC: Specificity
TO: Toe off
TN: True negative
TP: True positive
TPR: True positive rate
