# Supplementary figures and images for: Optimisation of a machine learning algorithm in human locomotion using principal component and discriminant function analyses
Source: PLoS One. 2017 Sep 8;12(9):e0183990. doi: 10.1371/journal.pone.0183990 (PMC5590884; doi:10.1371/journal.pone.0183990)

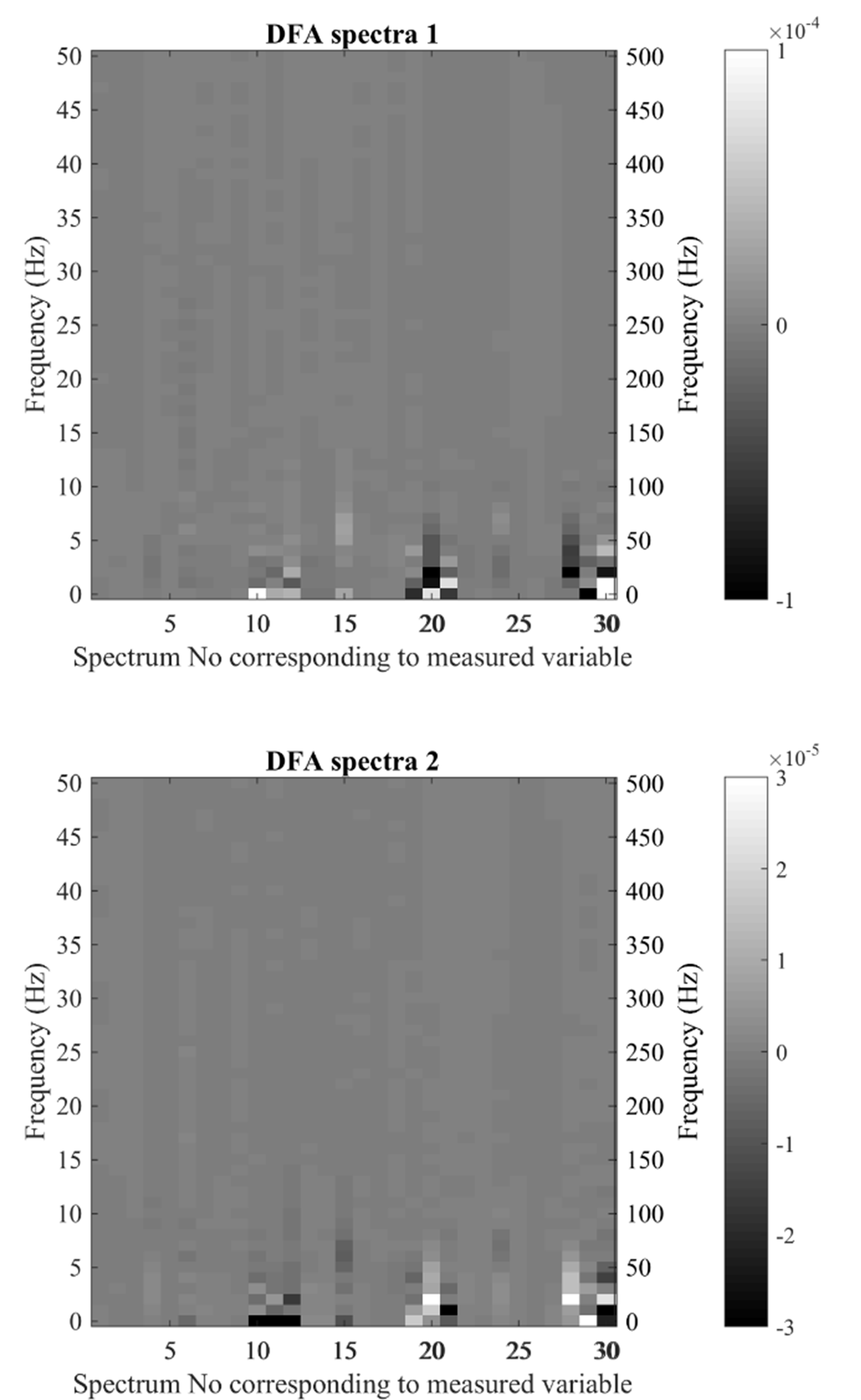

Supplement: S1 Fig — The right-hand side vertical axis is valid for variables 1 to 3, whilst the left-hand side one is valid for all remaining variables. Note that frequencies above 150 Hz do not contribute to the discrimination and the high importance of ultra-low frequencies. In some instances (e.g. variables No. 15 and 24), frequencies as high as 90 Hz contribute to the discrimination. In the main manuscript, the data shown in Fig 4. was obtained by integrating the absolute values of the spectra shown here, over all frequencies. The data shown in Fig 10. is obtained by cross-correlating the spectra shown here with the spectra coming from the raw variables (cross correlating any raw variable spectrum respectively with DF spectra 1 and 2 provide the corresponding DF score 1 and 2). (TIF) [file pone.0183990.s001.tif]

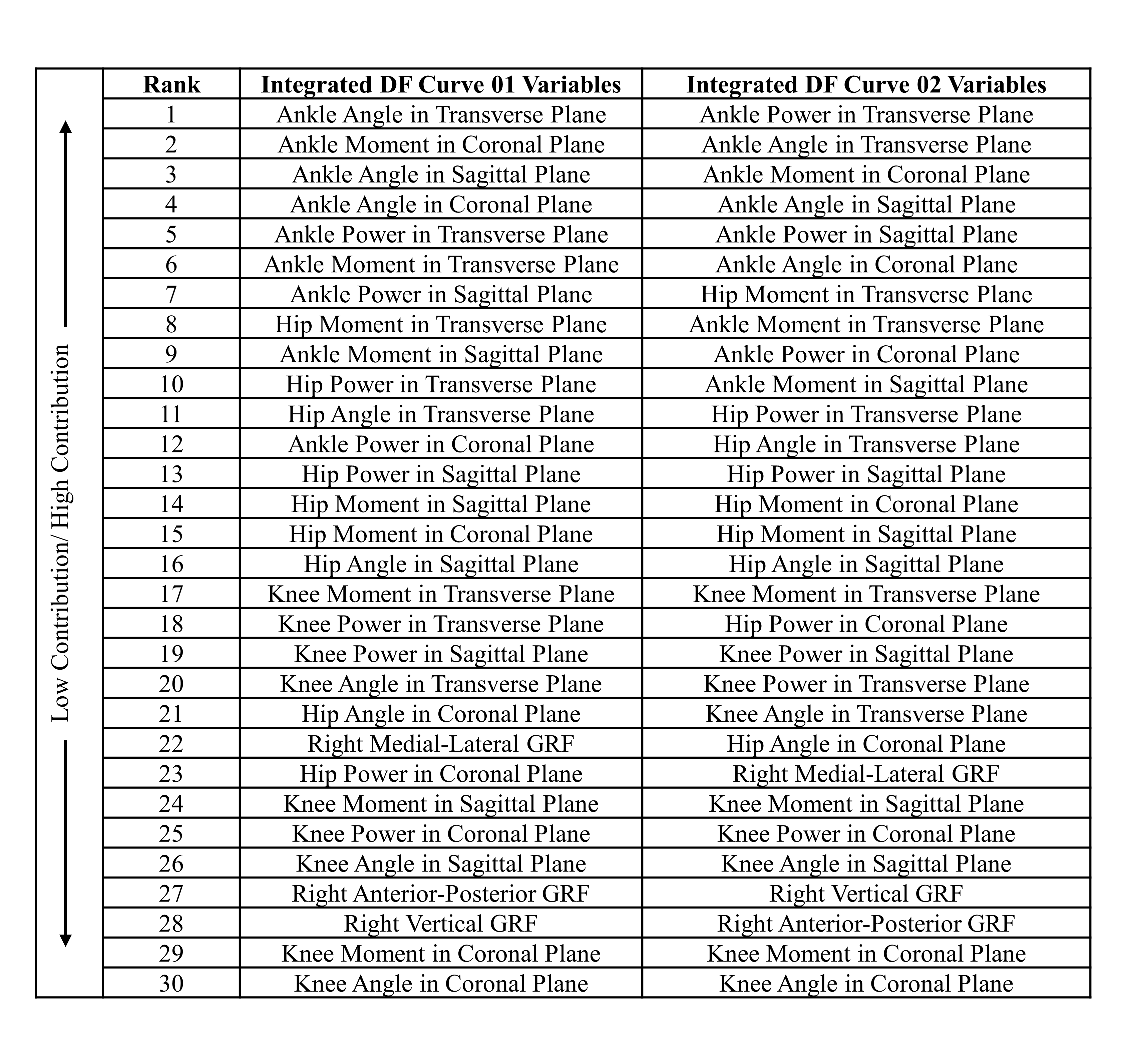

Supplement: S2 Fig — (TIF) [file pone.0183990.s002.tif]

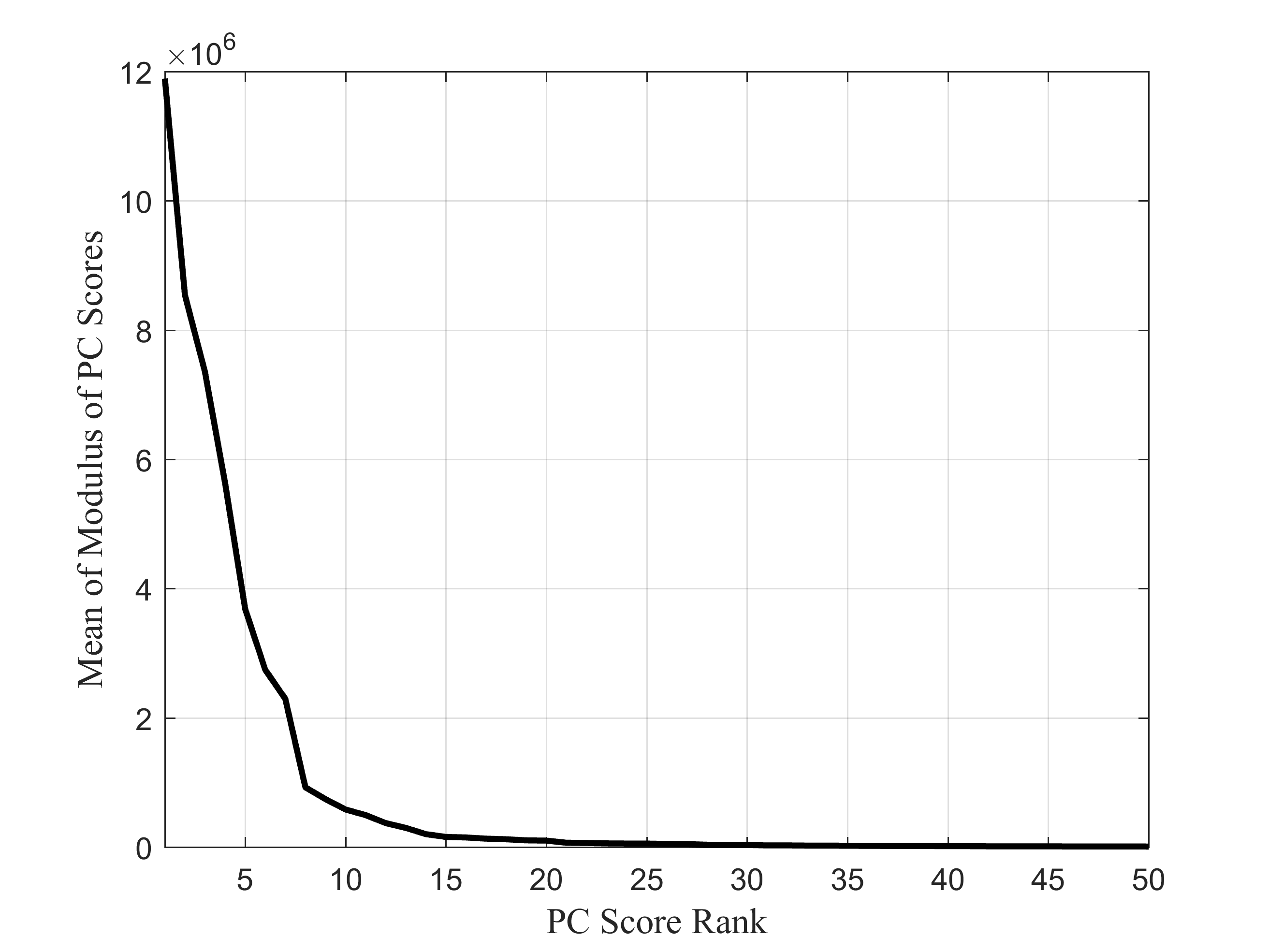

Supplement: S3 Fig — (TIF) [file pone.0183990.s003.tif]
